# Assessment of Age-Related Changes on Masticatory Function in a Population with Normal Dentition

**DOI:** 10.3390/ijerph18136899

**Published:** 2021-06-27

**Authors:** Seonhui Kim, Re-Mee Doh, Leegang Yoo, Sol-Ah Jeong, Bock-Young Jung

**Affiliations:** 1 Department of Dentistry, The Graduate School, Yonsei University, Seoul 03722, Korea; su24us@naver.com; 2 Department of Advanced General Dentistry, College of Dentistry, Dankook University, Cheonan 31116, Korea; remeedoh@dankook.ac.kr; 3 Department of Advanced General Dentistry, College of Dentistry, Yonsei University, Seoul 03722, Korea; ylgstory@naver.com (L.Y.); sola5256@naver.com (S.-A.J.)

**Keywords:** masticatory performance, masticatory function, age-related changes, food intake ability, posterior bite area

## Abstract

This study aimed to investigate the influence of changes in age-related physiological muscular and dental factors on masticatory function. This study was conducted in 211 healthy participants divided into four different age groups: 20–45 years (Gr1); 45–60 years (Gr2); 61–70 years (Gr3); and ≥71 years (Gr4). For objective evaluation of masticatory function, the masticatory performance, bite force, posterior bite area (PBA), functional tooth units (FTUs), the number of remaining teeth, tongue pressure, masseter muscle thickness (MMT), and handgrip strength were examined. Food intake ability (FIA) and the Oral Health Impact Profile-14 score were assessed subjectively using questionnaires. A significant decrease in the number of remaining teeth, FTUs, handgrip strength, and FIA was found in Gr4, and a significant decrease in the tongue pressure, PBA, and bite force was found in those aged ≥61 years. In groups 1 and 3, an association of the PBA with masticatory performance was observed. However, there was no significant decreasing trend in the MMT with respect to masticatory performance with aging. With sufficient FTUs and posterior tooth support, although age-dependent decreases in the bite force, tongue pressure and handgrip strength were observed, masticatory performance was maintained. Establishing the PBA by improving occlusion through dental treatment is thought to be important for masticatory function.

## 1. Introduction

It is commonly understood that muscle weakness occurring in the elderly population could reduce tongue pressure and lip motor function. Although tooth loss, which may be experienced during the life cycle, is a disease and pathological phenomenon, it is observed more frequently in older populations, and masticatory function is weakened sequentially due to aging [1].

Masticatory function can be representatively expressed in terms of masticatory performance and masticatory ability. The evaluation of masticatory function includes objective methods used to measure an individual’s capacity and subjective methods used to measure an individual’s response [2]. In the objective evaluation of masticatory function, factors including the number of remaining teeth, occlusal strength, and perioral muscles are considered as the indicators of static masticatory function. Meanwhile, as an organically linked dynamic process, mastication could be assessed as masticatory performance to measure mastication efficiency. Such methods used to evaluate masticatory performance include the sieving method to test comminution with natural [3] or artificial test food [4], the use of two-colored wax to test mixing [5], and the use of glucose gummies to test shearing [6]. Subjective evaluation of masticatory function is conducted using questionnaires asking about the kinds of chewable food or chewing ability [7]. The level of oral health-related satisfaction can be assessed by the oral health-related quality of life (OHRQoL) determined using the Oral Health Impact Profile (OHIP) questionnaire, which deals with self-perceived oral and general health [8].

Numerous previous studies regarding the dependence of masticatory performance on the dental status have demonstrated the following: First, dental factors influence masticatory function [2,9,10,11,12]. Second, the bite force and occluding area, which are influenced by the number of teeth and functional tooth units (FTUs), are correlated with masticatory performance [13]. Third, age per se has no influence on masticatory performance [14].

Recent studies have reported the relationship between masticatory function and the oral motor system. The amount of total muscle decreases by approximately 40% between the ages of 20 and 80 years; thus, people may experience a loss of functional motor units with advancing age [15]. In a study related to age-related changes in perioral muscle, it was demonstrated that tongue strength is positively related to handgrip strength [16]. A study based on 60 healthy young adults reported that the maximum tongue pressure influenced masticatory performance [17]. According to a study in more than 260 participants, including those who wore dentures, there was a significant correlation between masticatory performance and handgrip strength, while there was no correlation between masticatory performance and tongue pressure in elderly individuals with occlusal support [18]. Another study measured the self-assessed food intake ability (FIA) in 512 people who were ≥60 years old and reported a strong correlation between the dental status and subjective masticatory ability [19].

The relationship between the perioral muscle and masticatory function in terms of aging has been studied; however, the mechanism of action between the two has not yet been fully understood, since few studies have addressed both subjective and objective masticatory function. Therefore, it is necessary to conduct objective and subjective investigations simultaneously by considering the age-related physiological changes in not only dental factors but also perioral muscular factors.

It is hypothesized that both subjective and objective masticatory function decreases with the aging process of skeletal and muscular strength. The purpose of this study was to explore the differences between subjective and objective masticatory function in different age groups and to investigate the influence of changes in age-related physiological muscular and dental factors on masticatory function. As a cross-sectional study based on healthy dentate individuals, this study aimed to offer age-specific baseline data with clinical value for evaluating masticatory function.

## 2. Materials and Methods

### 2.1. Participants

This study was conducted in patients who visited the Department of Advanced General Dentistry at Yonsei University Dental Hospital between August 2020 and December 2020. Measurements were collected from 220 healthy and independent individuals, based on the calculation of sample size using G*power 3.1 software (Kiel University, Kiel, Germany) with α as 0.05, power as 0.8, and 0.25 of effect size [20,21]. Participants who were capable of independent social activities without any general illness and had at least 26 remaining teeth, including fixed dental prostheses, were selected for this study to minimize the influence of both the physical and dental status on the outcome of the masticatory function assessment. The exclusion criteria were as follows: a systemic disease, such as a disorder of saliva secretion or neuromuscular function; cognitive impairment; moderate to advanced periodontitis; or arch relationship with malocclusion or occlusal trauma. Of the 220 participants, nine individuals with incomplete data sets (6 participants from Gr 3 and 3 from Gr 4) were excluded. Finally, 211 volunteers who met the inclusion criteria for this study were divided into two major groups as adults (≤60 years) and elderly individuals (>60 years) and assigned to four different age groups, as follows: group 1, 20–45 years (*n* = 51; mean age: 32.1 ± 7.4); group 2, 46–60 years (*n* = 54; mean age: 53.5 ± 4.2); group 3, 61–70 years (*n* = 45; mean age: 66.2 ± 2.8); and group 4, 71 years or older (*n* = 61; mean age: 77.4 ± 5.2). Participants were recruited through voluntary registration and given informed consent for their participation.

### 2.2. Study Design

**Figure A1:**
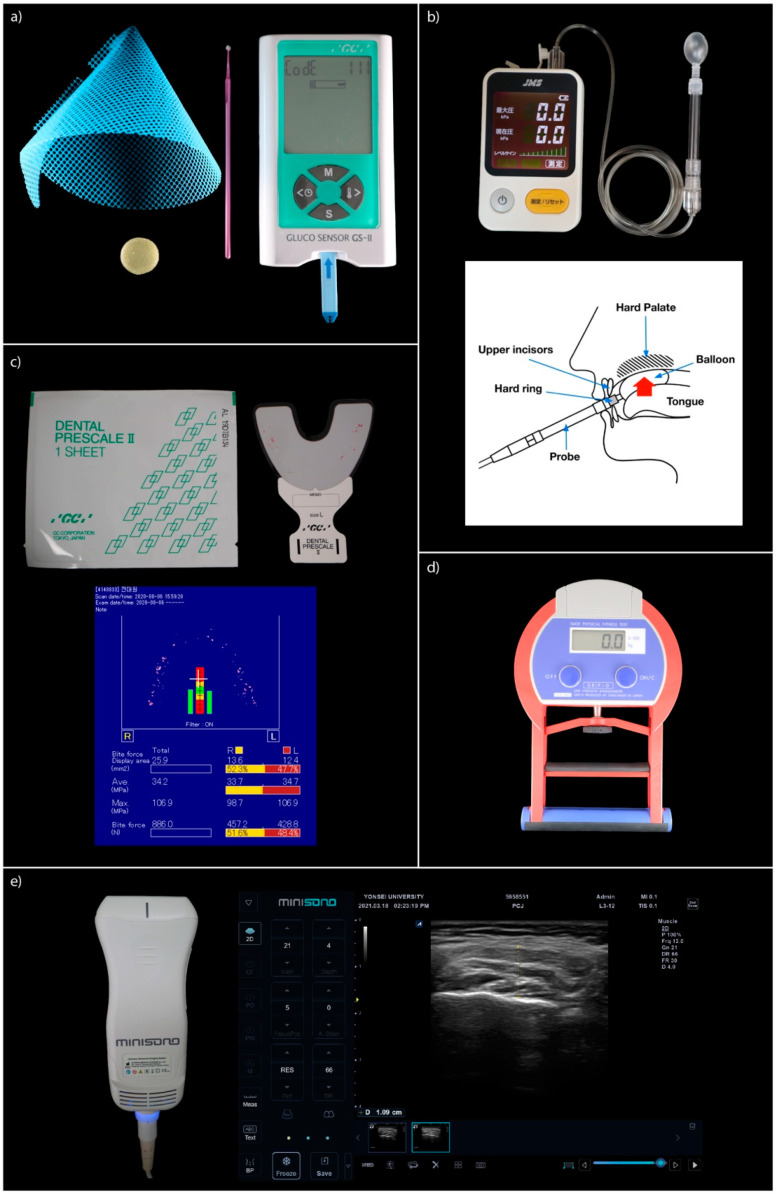
Measuring devices for objective assessment in this study. (**a**) masticatory performance, GS-2. (**b**) tongue pressure, TPM-01 [45]. (**c**) bite force and occluding bite area, Dental Prescale and Occluser software program. (**d**) handgrip strength, Takei handheld dynamometer. (**e**) masseter muscle thickness, Minisono.

Mastication function was assessed through objective and subjective measurements. The objective assessment of masticatory function was performed by evaluating the dental status through an oral examination and study model analysis, as well as examining dynamic and static masticatory function by measuring masticatory performance and dental status indicators, such as the number of FTUs and remaining teeth, tongue pressure, bite force, posterior bite area (PBA), handgrip strength, tooth wear and masseter muscle thickness (MMT) (see Figure A1). For the subjective assessment of masticatory function, FIA and OHRQoL were investigated using questionnaires. The whole process of all assessments was conducted by one researcher. The entire research protocol was conducted under the approval by Institutional Review Board of Yonsei University Dental Hospital (Approval number: 2–2020-0047).

#### 2.2.1. Objective Assessment

##### Dental Status

FTUs, the number of remaining teeth and tooth wear were examined. All participants belonged to Eichner index A, with four posterior support zones [22]. Tooth wear was determined by examining the tooth wear of the study model. When patterns of attrition, including facets, were observed in the region over one of the four quadrants, including (pre)molars. Tooth wear was coded as follows: tooth wear = 1; no tooth wear = 0. To prove intra-examiner reliability, the same examiner re-performed the assessment of tooth wear of 80 pairs of study models without referencing the prior data and the Cohen’s Kappa value was calculated as an excellent agreement (k = 0.822) [23]. FTUs were recorded by counting the number of occluding posterior tooth pairs. With the existence of a pair of opposing teeth in both the upper and lower arches, the molar area (excluding the 3rd molar) was recorded as 2 FTUs, and the premolar area was recorded as 1 FTU. Therefore, full dentition could also be indicated as 12 FTUs [9].

##### Masticatory Performance

Masticatory performance was determined by measuring the amount of dissolved glucose. For this test, a specifically designed 2 g-gummy containing dissolvable glucose (Gurucolum, GC, Tokyo, Japan) was used. The amount of dissolved glucose after the chewing exercise was measured using a specifically designed device (Glucosensor GS-2, GC, Tokyo, Japan), presenting the masticatory performance in numerical values. Participants were asked to chew the glucose-containing gummy for 20 s in their habitual chewing pattern, to rinse their mouth with 10 mL of purified water and to spit everything in their mouth, including the purified water, jelly, and saliva, onto a filter in a cup. The glucose-containing solution that was collected after filtering the comminuted jelly particles was mixed carefully with a brush and then coated onto the sensor chip. The value indicated by the measuring device was recorded.

##### Tongue Pressure

With regard to tongue pressure, although the forces applied during dynamic oral function for eating cannot be measured, the pressure loaded on the anterior region of the tongue has been used as an indicator of the tongue muscle strength with the consideration that it reflects the reserve capacity of the tongue [24]. The tongue pressure was measured using a probe (TPM-01, JMS, Hiroshima, Japan) consisting of a plastic catheter and a balloon. The participant was asked to sit on a chair, to hold the wing part of the probe connected to a balloon with his/her incisor teeth, to locate the balloon in the anterior palatal region, and to compress the balloon as powerfully as possible onto the palate for approximately 7 s. The measurement was repeated three times, and the mean value of the three records was used.

##### Bite Force and Occluding Bite Area

The bite force, which is the strength of force exerted from the coordination of the masticatory muscles and occluding teeth, was measured using a pressure-sensitive sheet (97 μm) [25]. The participant was asked to sit on a chair with the Frankfort horizontal plane parallel to the floor and the head in a relaxed state and to bite the sheet at the maximal inter-cuspal position with his/her maximum occlusal force for three seconds. The pressure measurement film was analyzed with a Bite force analyzer (Dental Prescale 50H, GC, Tokyo, Japan) [26,27]. In the analysis, the total bite force and the posterior and anterior occlusal contact areas were measured using Occluser 709 software (Occluser 709, GC, Tokyo, Japan).

##### Handgrip Strength

The handgrip strength has been used simply as an indicator of whole-body mass [28]. The participant was asked to stand up and to grab and squeeze the hand dynamometer (Takei handheld dynamometer, Japan) with his/her maximum power. The measurement was repeated three times for each hand, and the mean values were calculated and used.

##### Masseter Muscle Thickness (MMT)

The MMT is a useful index related to the strength of the masseter muscle [29], and it can be measured using an ultrasonic device. The values measured in the middle region during contraction of the left and right masseter muscles were used in this study, and the mean values of the measurements obtained from 3 points in the anterior, middle, and posterior regions of one cross-sectional image were used (Minisono, Alpinion, Anyang, Korea) [30]. Measurements were performed at the middle region, in which the outer fascia of the muscle and bony structure on the lateral side of the mandible are present, based on a report indicating that this site yields the best reproducibility [30,31].

#### 2.2.2. Subjective Assessment

##### Food Intake Ability (FIA) Questionnaire (14 Items)

**Figure 1:**
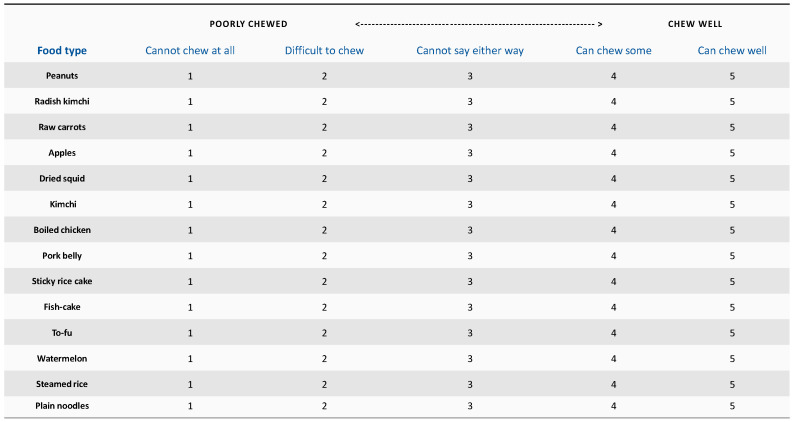
FIA questionnaire on 14 food items.

Masticatory ability was evaluated by asking the participants to complete a questionnaire listing 14 kinds of food ranging from soft and easy-to-chew food to hard food. Fourteen kinds of food included among the 30 kinds of food used in a previous study were included [25]. A five-point Likert scale was used, with a higher score indicating a better masticatory ability, as follows: 1 point (cannot chew at all); 2 points (difficult to chew); 3 points (cannot say either way); 4 points (can chew some); and 5 points (can chew well). (Figure 1).

##### OHIP-14

The OHIP questionnaire has been widely used as one of the most common questionnaires for evaluating self-satisfaction with OHRQoL. The OHIP-14 evaluates seven items, including functional limitation, pain, psychological discomfort, physical disability, psychological disability, social disability, and handicap, with a four-point Likert scale (0 = never, 1 = hardly ever, 2 = occasionally, 3 = fairly often, and 4 = very often). The lower the total score is, the better the OHRQoL [32].

### 2.3. Statistical Analysis

A normal data distribution for each variable in the age groups was confirmed using the Shapiro-Wilk test (see Table A1). One-way ANOVA and chi-square tests were used to investigate the significance of differences among age groups. The *p* for trends in masticatory function with increasing age was tested by simple linear regression or the Mantel-Haenszel chi-square test. Multiple comparisons were performed using Tukey’s test. Bivariate correlation coefficients were calculated to examine correlations among variables by Pearson’s correlation analysis. Multiple linear regression analysis was carried out to test the association of each explanatory variable with the outcome variable after controlling for the other variables. Variables associated with masticatory performance and FIA in the univariate analysis were chosen as independent variables for multivariate analysis (see Tables S1 and S2). A value of *p* < 0.05 was considered to indicate statistical significance. All data were analyzed using the statistical software R, version 4.0.4 (R Foundation for Statistical Computing, Vienna, Austria).

## 3. Results

### 3.1. Differences in Masticatory Function among Different Age Groups

**Figure A2:**
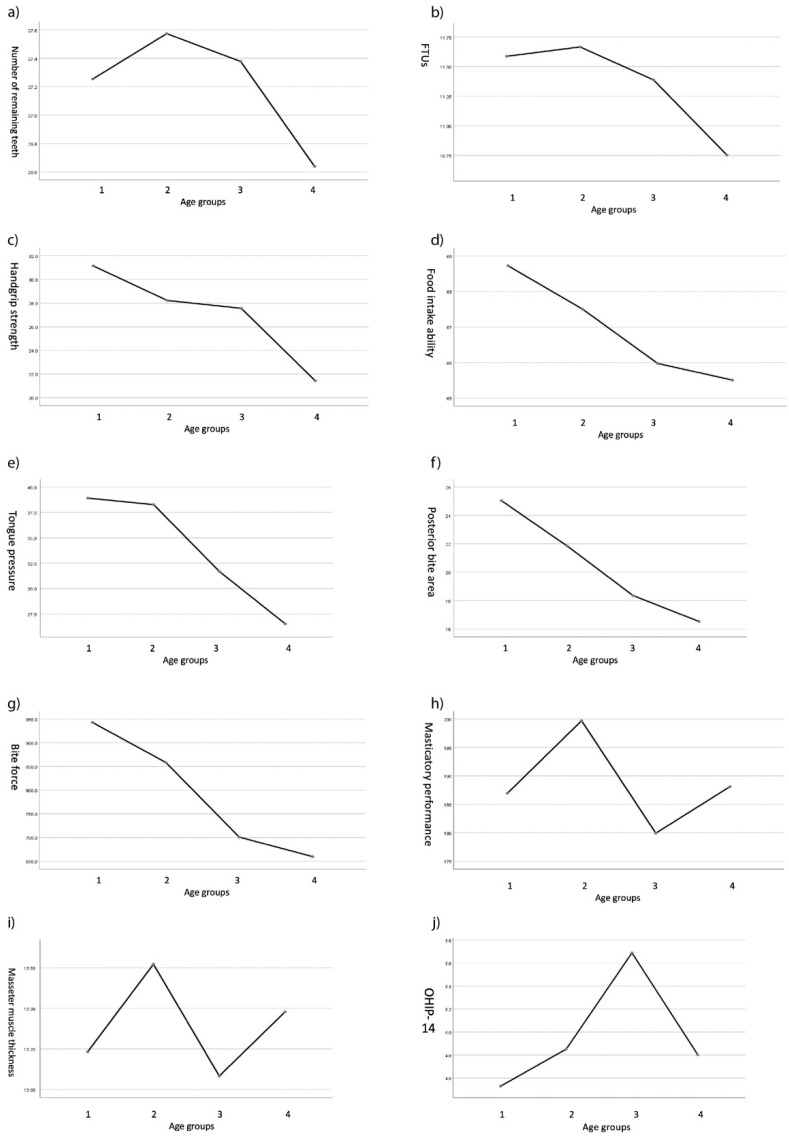
Changes of the variable in masticatory function among age groups. (**a**) the number of remaining teeth. (**b**) functional tooth units. (**c**) handgrip strength. (**d**) FIA. (**e**) tongue pressure. (**f**) PBA. (**g**) bite force. (**h**) masticatory performance. (**i**) MMT. (**j**) OHIP-14.

The dental status and demographic characteristics of each age group are presented in Table 1. The results of the analysis of masticatory function for each age group are shown in Table 2. The number of remaining teeth (*p* = 0.007), FTUs (*p* < 0.001), PBA (*p* < 0.001), bite force (*p* < 0.001), tongue pressure (*p* < 0.001), and handgrip strength (*p* < 0.001) significantly decreased with increasing age. There were significant decreases in the number of remaining teeth (*p* = 0.007), FTUs (*p* < 0.001), and handgrip strength (*p* < 0.001) in group 4. Significant decreases in tongue pressure (*p* < 0.001), PBA (*p* < 0.001), and bite force (*p* < 0.001) were observed in group 3, reaching the lowest value in group 4. The number of patients without tooth wear was significantly higher among group 1 than among those in the other age groups. The MMT, anterior bite area (ABA) and OHIP-14 score showed no significant differences in any age group. Masticatory performance, which is an objective assessment of masticatory function, presented no significant intergroup differences with increasing age, while FIA, which is a subjective assessment of masticatory function, showed a significantly decreasing trend with increasing age, reaching the lowest value in group 4. (see Figure A2).

### 3.2. Bivariate Correlations with Variables

The level of correlation was interpreted based on the correlation coefficient, as follows: negligible (0–0.3); weak (0.3–0.5); moderate (0.5–0.7); strong (0.7–0.9); and very strong (≥0.9) [33]. In all age groups, a significant, strong positive correlation was found between the number of remaining teeth and FTUs (Gr1: r = 0.96, Gr2: r = 0.87, Gr3: r = 0.94, and Gr4: r = 0.89, *p* < 0.001, respectively) and between the PBA and bite force (Gr1: r = 0.73, Gr2: r = 0.96, Gr3: r = 0.93, and Gr4: r = 0.84, *p* < 0.001, respectively). In addition, a significant, weak to moderate positive correlation was found between the MMT and bite force (Gr1: r = 0.45, Gr2: r = 0.48, Gr3: r = 0.44, and Gr4: r = 0.56, *p* < 0.01, respectively), between the MMT and PBA (Gr1: r = 0.52, Gr2: r = 0.45, Gr3: r = 0.45, and Gr4: r = 0.53, *p* < 0.01, respectively), and between the handgrip strength and tongue pressure (Gr1: r = 0.56, Gr2: r = 0.43, Gr3: r = 0.53, and Gr4: r = 0.47 *p* < 0.01, respectively) in all age groups. The MMT and handgrip strength showed a decreasing correlation with increasing age (Gr1: r = 0.74, Gr2: r = 0.52, Gr3: r = 0.54, and Gr4: r = 0.43, *p* < 0.01, respectively), indicating a strong to weak correlation with significance. Only group 3 presented a significant, strong correlation (r = −0.8) of FIA with the OHIP-14 score, while the other groups showed a weak negative correlation with significance (Gr1: r = −0.45, Gr2: r = −0.42, and Gr4: r = −0.49, *p* < 0.01, respectively). A significant correlation of age with dental factors and muscle factors was only observed in group 4, and age and tongue pressure showed a significant, moderate negative correlation (r = −0.5, *p* < 0.01). (Table 3).

### 3.3. Multiple Linear Regression Analysis with Masticatory Performance

In groups 1 and 3, the PBA (Gr1 and Gr3: *p* = 0.044) was significantly associated with masticatory performance. In groups 2 and 4, however, no significant relationship was found between masticatory performance and any of the independent variables. The OHIP-14 score (*p* = 0.014) was also significantly associated with masticatory performance in group 1. (Table 4).

### 3.4. Multiple Linear Regression Analysis with Food Intake Ability

In all age groups, a significant association between FIA and the OHIP-14 score (*p* < 0.001, respectively) was observed. In addition, a significant relationship was found with FTUs (*p* = 0.002) in group 2 and with tongue pressure (*p* = 0.047) in group 3. (Table 5).

### 3.5. Stepwise Multiple Linear Regression Analysis with Handgrip Strength

In all age groups, tongue pressure (Gr1: *p* = 0.002, Gr2: *p* = 0.024, Gr3: *p* = 0.002, and Gr4: *p* < 0.001) was significantly associated with handgrip strength. Moreover, a significant relationship was observed with the MMT (Gr1: *p* < 0.001; Gr2 and Gr3: *p* = 0.001) in all age groups except group 4. (Table 6).

## 4. Discussion

In this study, the influence of oral motor system and dental factors on the masticatory function on the basis of aging were investigated and the difference between subjective and objective masticatory function in different age groups was explored. To our knowledge, this is the first study to address this subject.

A trend of significant decreases in tongue pressure (*p* < 0.001) and handgrip strength (*p* < 0.001) with aging was observed in this study. An obvious decrease in tongue pressure was found in groups 3 and 4, whereas an obvious decrease in handgrip strength was seen in group 4 compared with the other groups. A significant correlation between handgrip strength and tongue pressure in all age groups was found in this study (Gr1: r = 0.56, Gr2: r = 0.43, Gr3: r = 0.53, and Gr4: r = 0.47, *p* < 0.01, respectively). In group 4, a moderate correlation between age and tongue pressure (r = −0.5) and a weak correlation between age and handgrip strength (r = −0.31) were found. This is consistent with the results reported in previous studies. Human age-related muscle atrophy begins at approximately the age of 25 years and accelerates after approximately the age of 60 [34]. Likewise, in the tongue muscle, fatty infiltration and amyloid deposition increase with aging, resulting in a decrease in muscle fibers and ultimately a decrease in tongue pressure [28]. Hara et al. conducted age-specific research in 980 people, including denture wearers, and reported very similar patterns of reduction in tongue pressure and handgrip strength with aging in both sexes and more drastic reductions in the elderly group than in the adult group. Tongue pressure has been considered to be influenced by skeletal muscle mass and muscle power in adults [34]; hence, changes in perioral muscle, such as those indicated by changes in tongue pressure, could potentially be predicted using handgrip strength, which is easy to measure.

Some previous studies have reported tongue pressure as a significant factor in measuring masticatory performance [17,35]. Another study reported no association of tongue pressure with masticatory performance [18], which is consistent with the results of this study. Despite similarities in the dental status, the different results among these studies could be due to the different assessment methods and subject selection criteria. The present study adopted the assessment of shearing ability as a method for measuring masticatory performance. According to Yamada et al.’s study [36] on masticatory performance in young dentate adults, there was no significant correlation between shearing ability and tongue function, but masticatory performance assessed using shearing ability showed a significant correlation with both the occlusal force and occlusal contact area, which are results similar to those of the present study.

Meanwhile, a previous study on the relation between oral motor function and masticatory performance in elderly individuals highlighted the importance of tongue function in masticatory performance by using a color-changeable chewing gum to assess the masticatory performance [37]. Measurement of mixing ability assisted by tongue and lip function can allow a more efficient analysis of masticatory performance in elderly individuals with decreased tongue pressure and clarify the relationship between tongue pressure and masticatory performance.

The correlation between the handgrip strength and MMT decreased from strong (r = 0.736) in group 1 to weak (r = 0.425) in group 4, and no significant change was observed in the intergroup comparison of the MMT. This result is interpreted to indicate the value of the handgrip strength, which represents the decreasing strength of muscles in general with aging, while the MMT is maintained throughout life in subjects with normal dentition. If only considering the result of the Pearson correlation analysis, it might be concluded that the handgrip strength can be assessed instead of the force exerted by the oral musculature. As an additional analysis, multiple linear regression analysis with the handgrip strength indicated that the handgrip strength method could be substituted with the tongue pressure and MMT in subjects <71 years old (*p* < 0.001), whereas in group 4, it could be substituted with the tongue pressure (*p* < 0.001) but not the MMT.

The bite force was decreased (*p* < 0.001) with aging, and a significant decrease was found in those aged ≥61 years. The significant correlation between the MMT and bite force in all subjects (r = 0.456, *p* < 0.001) (see Table A2) is consistent with the result of a previous study reporting a significant association (r = 0.41, *p* < 0.001) between the cross-section of the masseter muscle and bite force. That study also reported the masseter muscular strength as a major contributing factor of bite force in adults [3]. One hypothesis in this study was that the bite force is positively related to the MMT and that these two variables decrease with aging; however, it was rejected, as the change in the MMT with aging was not significant (*p* = 0.72). The reason why the MMT did not decrease with aging decreases in bite force can be deduced. The masseter muscle predominantly consists of type 1 fibers, which are mainly associated with masseter muscle atrophy caused by muscle disuse [29,38]. The elderly participants in this study retained a sufficient number of functioning teeth; therefore, the influence of disuse due to tooth loss did not lead to atrophy of the masseter muscle or physiological muscle changes with aging. Previous studies have reported that the thickness of the masseter muscle is reduced by tooth loss [29], with edentulous patients showing significantly thinner masseter muscles than dentate patients [39]. Under edentate conditions, therefore, the MMT might be decreased by reduced masticatory function.

The PBA can be considered as a factor associated with bite force. A strong correlation was found between the PBA and bite force in this study (r range: 0.73–0.96). Previous studies directly comparing the PBA and bite force could not be found; most studies have compared each of them with masticatory performance. Ikebe el al. reported that the association of bite force with masticatory performance was significant and that the decrease in masticatory performance was significant in Eichner index B rather than in Eichner index A, suggesting the importance of the posterior occlusal contact of the remaining dentition [40].

In the regression analysis of masticatory performance, a significant association with the PBA rather than bite force was found in groups 1 and 3. In a previous study, where masticatory performance was measured using the sieving method in 30 patients with 28 teeth, the occlusal contact area was reported as the most pivotal determinant of masticatory performance, followed by the maximum bite force [41]. The authors also reported better masticatory performance with larger occlusal contact areas and stronger premolar bite force. In this study, with participants retaining sufficient FTUs over the threshold level [10] affecting masticatory function, the area of occlusion, such as the PBA, had more influence on masticatory performance than the bite force.

An extremely high (r range: 0.87–0.96) correlation was found between the number of remaining teeth and FTUs. In addition, a trend of a significant decrease with aging was observed in the number of remaining teeth (*p* = 0.007) and FTUs (*p* < 0.001). However, the participants of this study were limited to healthy dentate patients, and a difference in the number of teeth <1 was considered to be a small difference; hence, the number of teeth was not considered to cause any significant difference in the results or play a role as a confounding factor in this study. Hatch et al. stated that the number of FTUs and bite force influenced masticatory performance and that the number of FTUs was the most crucial factor [3]. Many studies have suggested that >20 teeth and >8 FTUs are sufficient to maintain masticatory function and that <5 FTUs is the threshold for indicating a problematic dental functional status [2,10,14,19,42]. Because the inclusion criteria of this study were over the threshold suggested above (26 teeth and 10 FTUs on average), it was inferred that there was no change in masticatory performance with aging. Statistically, the results also showed that masticatory performance was not associated with age as an independent variable in the regression analysis in any age group. In conclusion, with a good dental status, such as a sufficient number of FTUs, although age-dependent decreases in muscular factors, such as tongue pressure and handgrip strength, and occlusal factors, such as PBA and bite force, were also observed, masticatory performance was maintained with aging. Establishment of the occlusal contact area could thus be important for improving masticatory performance through restorative dental treatment.

The PBA obtained by quantifying the posterior occlusal area and tooth wear was analyzed with respect to masticatory performance among the age groups. With aging, the rate of tooth wear increased, but the PBA decreased, whereas no change in masticatory performance was observed. By regarding the tooth wear parameter as a characteristic of the increased occlusal area of worn teeth, it was investigated whether an increase in tooth wear can lead to an increase in masticatory performance. In the univariate regression analysis in this study, none of the age groups presented a significant relation between the influence of tooth wear and masticatory performance. This is consistent with the result of a recent report on the effect of tooth wear on masticatory performance, suggesting no significant relationship between tooth wear severity in the post-canine area and masticatory performance [43]. Hence, tooth wear was not considered to have an influence on masticatory performance in this study. However, tooth wear was assessed as a nominal variable, as the area of the wear itself could not be analyzed as a continuous variable. In future studies, a continuous parametrization of the degree of tooth wear using a tooth wear index will be helpful for obtaining a clear understanding.

Food intake ability (FIA), as a parameter of the subjective masticatory function evaluation, significantly decreased with aging, which is different from the trend in masticatory performance, which did not present a significant change in the objective masticatory function evaluation. However, similar to masticatory performance, an association between FIA and age was not found on regression analysis in any age group. Furthermore, a correlation between masticatory performance and FIA was not observed in any of the age groups, which is also consistent with the results of previous studies that have reported either almost no or a very weak correlation between the self-assessed masticatory ability and masticatory performance [2]. Masticatory function can be subjectively evaluated using combined factors, such as physical, psychological, and social factors; hence, it may be difficult to obtain results that are consistent with those of objective evaluations.

As a subjective assessment, the OHIP can be used to evaluate dental health from the view of the patient, and a high OHRQoL can reflect a favorable dental environment with high-quality dental prostheses or dentures. In the multiple linear regression, a significant relationship between FIA and the OHIP-14 score was found in all age groups in this study (*p* < 0.001). Similarly, Fueki et al. compared the OHIP score and patients’ perception of chewing ability assessed using a questionnaire on food intake and reported a significant, moderate relation between them [44]. Meanwhile, FIA showed inconsistent relationships with other variables, such as significant associations with the number of FTUs in group 2 and tongue pressure in group 3. It can be inferred that other factors not investigated in this study may affect FIA.

This study can provide the age-specific baseline data for clinical use regarding masticatory function. However, there are several limitations such as sample size, uneven gender distribution, and in particular difficulty in selecting participants due to poly-medication in the elderly over 60 years in this study. Longitudinal studies based on a larger number of participants are recommended to avoid sample size bias and to generalize the relations among the parameters with respect to masticatory performance and FIA. Investigations on the correlation between the PBA and tooth wear as well as on the perioral musculature function reflecting tongue and lip movement are needed for further research.

## 5. Conclusions

**Figure 2:**
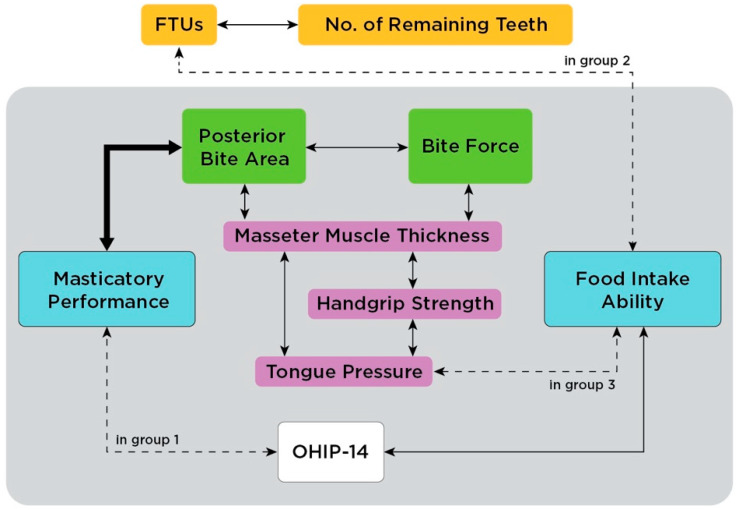
Summary of the significant interrelationships in this study among various factors of masticatory function.

This study demonstrates that masticatory function is related to many factors (Figure 2). Static masticatory function, except for the masseter muscle thickness (MMT), decreased with aging. However, a decrease in masticatory performance with aging was not observed, and the influence of tongue pressure on food intake ability (FIA) could not be generalized. From the perspective of subjective masticatory function, FIA was associated with OHIP-14 score, which was related to OHRQoL. Even if age-related factors, including the number of remaining teeth, posterior bite area (PBA), handgrip strength, and tongue pressure, decreased with aging, dynamic masticatory function, i.e., masticatory performance, was maintained throughout life as long as the PBA was established. In conclusion, the establishment of posterior occlusal support is important in oral rehabilitation for masticatory function in the elderly.

## Figures and Tables

**Table 1:** Demographic characteristics and dental status of the participants.

		Total	20–45 (1)	46–60 (2)	61–70 (3)	71+ (4)	*p*
Subjects N (%)		211 (100)	51 (24.2)	54 (25.6)	45 (21.3)	61 (28.9)	
Age		58 ± 17.9	32.1 ± 7.4	53.5 ± 4.2	66.2 ± 2.8	77.4 ± 5.2	
Gender (%) ^a^	male	95 (45.0)	25 (49.0)	26 (48.1)	21 (46.7)	23 (37.7)	0.591
	female	116 (55.0)	26 (51.0)	28 (51.9)	24 (53.3)	38 (62.3)	

^a^: Chi-square test.

**Table 2:** Assessment of the variables of masticatory function among age groups.

		Adult Group		Elderly Group		*p*	*p* for Trend ^b^	*Posthoc*
		20–45 (1)	46–60 (2)	61–70 (3)	71+ (4)			
Number of Remaining teeth		27.25 ± 1.59	27.57 ± 1.06	27.38 ± 1.13	26.64 ± 1.45	0.002 *	0.007 *	2, 3 > 4
FTUs		11.59 ± 0.80	11.67 ± 0.73	11.39 ± 1.09	10.75 ± 1.25	<0.001 *	<0.001 *	1, 2, 3 > 4
Masticatory performance (mg/dL)		186.94 ± 55.85	199.70 ± 68.37	179.93 ± 53.63	188.11 ± 58.22	0.415	0.674	
Tongue Pressure (kPa)		38.92 ± 10.93	38.28 ± 10.50	31.68 ± 7.82	26.52 ± 9.74	<0.001 *	<0.001 *	1, 2 > 3 > 4
Handgrip strength (Kg)		31.14 ± 10.49	28.22 ± 8.71	27.56 ± 9.29	21.44 ± 8.03	<0.001 *	<0.001 *	1, 2, 3 > 4
Posterior bite area (mm^2^)		25.03 ± 12.87	21.84 ± 12.16	18.36 ± 10.45	16.54 ± 8.02	<0.001 *	<0.001 *	1, 2 > 3,4
Anterior bite area (mm^2^)		3.14 ± 2.38	4.34 ± 5.89	5.51 ± 17.36	3.43 ± 3.72	0.548	0.794	
Bite force (N)		942.95 ± 443.82	858.41 ± 432.72	700.93 ± 416.20	659.85 ± 308.29	0.001 *	<0.001 *	1, 2 > 3,4
Masseter m. thickness (mm)		13.19 ± 2.67	13.62 ± 2.65	13.07 ± 2.18	13.38 ± 2.60	0.717	0.959	
Food intake ability		68.73 ± 2.93	67.50 ± 4.89	65.98 ± 5.99	65.51 ± 6.40	0.007 *	0.001 *	1 > 4
OHIP-14		4.53 ± 6.33	4.85 ± 8.21	5.69 ± 7.47	4.80 ± 6.13	0.870	0.744	
Tooth wear (%) ^a^	no	49 (96.1)	46 (85.2)	18 (40.0)	16 (26.2)	<0.001 *	<0.001 *	
	yes	2 (3.9)	8 (14.8)	27 (60.0)	45 (73.8)			

mean ± SD, * *p* < 0.05. ^a^: Chi-square test, ^b^: *p* for trend by simple linear regression or Mantel-Haenzel chi-square test.

**Table 3:** Pearson’s correlation coefficient in each age group.

Group		Age	RT	FTUs	MP	TP	HG	PBA	ABA	BF	MMT	FIA	OHIP-14
20–45 (1)	Age	1	−0.010	0.038	0.229	0.237	0.180	0.158	−0.052	−0.021	0.354 *	0.073	0.033
	RT		1	0.962 ***	−0.172	0.086	0.071	0.310 *	0.238	0.275	0.031	−0.097	0.219
	FTUs			1	−0.091	0.042	0.032	0.339 *	0.181	0.297 *	0.027	−0.108	0.146
	MP				1	−0.187	0.108	0.299 *	−0.033	0.261	0.174	0.128	−0.356 *
	TP					1	0.559 ***	0.135	0.226	0.029	0.402 **	0.068	0.038
	HG						1	0.455 **	0.113	0.432 **	0.736 ***	0.327 *	0.018
	PBA							1	0.118	0.730 ***	0.519 ***	0.194	−0.092
	ABA								1	0.246	0.105	0.153	0.267
	BF									1	0.449 **	0.229	−0.034
	MMT										1	0.291 *	0.083
	FIA											1	−0.449 **
	OHIP14												1
46–60 (2)	Age	1	0.137	0.082	0.014	−0.077	0.106	0.054	0.172	0.034	0.098	−0.126	−0.058
	RT		1	0.872 ***	0.301 *	−0.085	0.073	0.085	0.108	0.037	0.041	0.268 *	0.132
	FTUs			1	0.284 *	−0.032	0.014	0.202	0.118	0.166	0.029	0.447 **	0.084
	MP				1	0.354 **	0.171	0.412 **	−0.176	0.420 **	0.311 *	0.230	−0.030
	TP					1	0.439 **	0.342 *	−0.114	0.409 **	0.372 **	0.248	−0.048
	HG						1	0.261	−0.165	0.375 **	0.522 ***	0.129	−0.133
	PBA							1	−0.269 *	0.961 ***	0.452 **	0.251	−0.167
	ABA								1	−0.186	−0.326 *	0.174	−0.163
	BF									1	0.477 ***	.315 *	−0.232
	MMT										1	0.023	−0.079
	FIA											1	−0.422 **
	OHIP14												1
61–70 (3)	Age	1	−0.271	−0.256	−0.103	−0.016	−0.096	−0.135	−0.109	−0.207	0.139	−0.216	0.152
	RT		1	0.943 ***	0.169	0.090	0.009	0.032	0.079	−0.047	−0.197	0.199	−0.074
	FTUs			1	0.194	0.050	−0.038	0.084	0.078	−0.021	−0.201	0.227	−0.138
	MP				1	0.038	0.036	0.301 *	−0.233	0.268	0.059	0.188	−0.139
	TP					1	0.531 ***	0.095	−0.085	0.180	0.307 *	0.454 **	−0.291
	HG						1	0.183	−0.098	0.205	0.541 ***	0.370 *	−0.282
	PBA							1	−0.220	0.926 ***	0.451 **	0.139	−0.043
	ABA								1	−0.175	−0.162	−0.036	0.082
	BF									1	0.442 **	0.175	−0.069
	MMT										1	0.280	−0.137
	FIA											1	−0.797 ***
	OHIP14												1
71+ (4)	Age	1	−0.391 **	−0.304 *	0.034	−0.505 ***	−0.316 *	−0.322 *	0.354 **	−0.156	−0.247	−0.010	0.174
	RT		1	0.891 ***	0.068	0.032	0.162	0.396 **	−0.122	0.284 *	0.139	0.135	−0.250
	FTUs			1	−0.002	−0.050	0.104	0.392 **	−0.238	0.257 *	0.079	0.177	−0.225
	MP				1	0.039	0.085	0.203	0.106	0.161	0.138	0.136	−0.117
	TP					1	0.469 ***	0.428 **	−0.103	0.329 **	0.306 *	0.061	−0.112
	HG						1	0.568 ***	−0.076	0.466 ***	0.425 **	0.274 *	−0.234
	PBA							1	−0.115	0.835 ***	0.531 ***	0.228	−0.247
	ABA								1	0.270 *	0.217	0.126	−0.223
	BF									1	0.556 **	0.208	−0.293 *
	MMT										1	0.166	−0.304 *
	FIA											1	−0.491 ***
	OHIP14												1

*p* *** < 0.001, *p* ** < 0.01, *p* * < 0.05. RT: number of remaining teeth, FTUs: functional tooth units, MP: masticatory performance, HG: handgrip strength, PBA: posterior bite area, ABA: anterior bite area, BF: bite force, MMT: masseter muscle thickness, FIA: food intake ability, TP: tongue pressure.

**Table 4:** Multiple linear regression analysis of factors associated with masticatory performance.

		Multivariate				
		B	β	95% CI	*p*	R^2^
20–45 (1)	Age					
	Gender (M)					
	Gender (F)					
	RT					
	FTUs					
	TP					
	HG					
	PBA	1.164	0.268	0.032–2.296	0.044 *	0.198
	ABA					
	BF					
	MTM					
	FIA					
	OHIP-14	−2.926	−0.332	−5.229–−0.623	0.014 *	
	Tooth wear					
45–60 (2)	Age					
	Gender (M)					
	Gender (F)					
	RT	−1.848	−0.043	−15.495–11.800	0.790	0.106
	FTUs	1.015	0.018	−16.639–18.669	0.910	
	TP	−0.170	−0.032	−0.943–0.603	0.664	
	HG					
	PBA	1.146	0.219	−0.308–2.599	0.122	
	ABA					
	BF	0.012	0.087	−0.026–0.051	0.527	
	MTM	0.425	0.018	−3.724–4.574	0.840	
	FIA					
	OHIP-14					
	Tooth wear					
61–70 (3)	Age					
	Gender (M)					
	Gender (F)					
	RT					
	FTUs					
	TP					
	HG					
	PBA	1.547	0.301	0.042–3.053	0.044 *	0.091
	ABA					
	BF					
	MTM					
	FIA					
	OHIP-14					
	Tooth wear					
71+ (4)	Age					
	Gender (M)					
	Gender (F)					
	RT					
	FTUs					
	TP					
	HG					
	PBA					
	ABA					
	BF					
	MTM					
	FIA					
	OHIP-14					
	Tooth wear					

B: partial regression coefficient; β: standardized partial regression coefficient; * *p* < 0.05. RT: number of remaining teeth, FTUs: functional tooth units, FIA: food intake ability, HG: handgrip strength, PBA: posterior bite area, ABA: anterior bite area, BF: bite force, MMT: masseter muscle thickness, TP: tongue pressure.

**Table 5:** Multiple linear regression analysis of factors associated with FIA.

		Multivariate				
		B	β	95% CI	*p*	R^2^
20–45 (1)	Age					
	Gender (M)					
	Gender (F)					
	RT					
	FTUs					
	TP					
	HG	0.057	0.203	−0.043–0.156	0.257	0.329
	PBA					
	ABA					
	BF					
	MTM	0.198	0.180	−0.194–0.589	0.315	
	MP					
	OHIP-14	−0.216	−0.468	−0.328–−0.104	<0.001 *	
	Tooth wear					
45–60 (2)	Age					
	Gender (M)					
	Gender (F)					
	RT					
	FTUs	4.378	0.656	1.758–6.997	0.002 *	0.447
	TP					
	HG					
	PBA					
	ABA					
	BF	0.001	0.107	−0.001–0.004	0.356	
	MTM					
	MP					
	OHIP-14	−0.262	−0.440	−0.394–−0.130	<0.001 *	
	Tooth wear					
61–70 (3)	Age					
	Gender (M)					
	Gender (F)					
	RT					
	FTUs					
	TP	0.168	0.220	0.002–0.335	0.047 *	0.691
	HG	0.027	0.041	−0.150–0.203	0.761	
	PBA					
	ABA					
	BF					
	MTM					
	MP					
	OHIP-14	−0.573	−0.715	−0.732–−0.414	<0.001 *	
	Tooth wear					
71+ (4)	Age					
	Gender(M)					
	Gender(F)	−3.674	−0.280	−7.627–0.279	0.068	0.310
	RT					
	FTUs					
	TP					
	HG	−0.020	−0.025	−0.265–0.226	0.872	
	PBA					
	ABA					
	BF					
	MTM					
	MP					
	OHIP-14	−0.483	−0.462	−0.720–−0.246	<0.001 *	
	Tooth wear					

B: partial regression coefficient; β: standardized partial regression coefficient; * *p* < 0.05. RT: number of remaining teeth, FTUs: functional tooth units, MP: masticatory performance, HG: handgrip strength, PBA: posterior bite area, ABA: anterior bite area, BF: bite force, MMT: masseter muscle thickness, TP: tongue pressure.

**Table 6:** Stepwise multiple linear regression analysis of handgrip strength in each group.

Group	Dependent Variable	Independent Variable	B	S.E	β	*p*-Value	F (*p*-Value)	R^2^
1	HG	MMT	2.397	0.380	0.610	0.000	39.838 (*p* < 0.001)	0.624
		TP	0.301	0.093	0.314	0.002		
2	HG	MMT	1.370	0.402	0.417	0.001	13.273 (*p* < 0.001)	0.342
		TP	0.235	0.101	0.284	0.024		
3	HG	MMT	1.781	0.517	0.418	0.001	16.513 (*p* < 0.001)	0.440
		TP	0.479	0.144	0.403	0.002		
4	HG	TP	0.228	0.094	0.277	0.018	18.189 (*p* < 0.001)	0.385
		PBA	0.450	0.114	0.450	0.000		

B: unstandardized partial regression coefficient, β: standardized partial regression coefficient, which indicates the relative importance of each variable. HG: handgrip strength, PBA: posterior bite area, MMT: masseter muscle thickness, TP: tongue pressure.

## Supplementary Materials

The following are available online at https://www.mdpi.com/article/10.3390/ijerph18136899/s1, Table S1: Multiple linear regression analysis of factors associated with masticatory performance, Table S2: Multiple linear regression analysis of factors associated with food intake ability

## Appendix A

**Table A1:** The normality tests.

Age Groups		Shapiro-Wilk	Skewness	Kurtosis
		*p*		
20–45 (1)	PBA	0.180	1.002	1.031
	ABA	0.596	2.205	5.675
	RT	0.000	−1.441	0.670
	FTUs	0.000	−1.499	0.307
	MP	0.776	0.158	−0.487
	BF	0.871	0.717	−0.248
	TP	0.571	−0.399	0.263
	HG	0.004	0.166	−1.063
	MMT	0.284	0.000	−0.705
	FIA	0.000	−2.958	9.787
	OHIP-14	0.000	1.119	−0.217
46–60 (2)	PBA	0.162	0.628	0.206
	ABA	0.060	4.648	23.767
	RT	0.000	−1.945	4.665
	FTUs	0.000	−2.211	4.066
	MP	0.300	0.235	−0.739
	BF	0.068	0.541	−0.299
	TP	0.621	−0.281	−0.137
	HG	0.054	0.140	−1.058
	MMT	0.542	0.186	−0.709
	FIA	0.000	−2.517	5.455
	OHIP-14	0.000	2.612	7.587
61–70 (3)	PBA	0.014	0.992	1.106
	ABA	0.000	6.605	44.055
	RT	0.000	−2.276	5.426
	FTUs	0.000	−1.896	3.124
	MP	0.404	0.521	0.539
	BF	0.905	1.088	1.619
	TP	0.354	−0.499	0.805
	HG	0.342	0.085	−1.300
	MMT	0.372	0.430	0.316
	FIA	0.000	−1.765	2.114
	OHIP-14	0.354	1.436	1.257
71+ (4)	PBA	0.681	0.128	−0.423
	ABA	0.000	2.899	9.808
	RT	0.000	−0.491	−0.329
	FTUs	0.000	−0.740	−0.102
	MP	0.662	−0.154	−0.014
	BF	0.489	0.017	−0.446
	TP	0.360	0.187	1.235
	HG	0.197	0.336	−0.404
	MMT	0.242	0.297	0.401
	FIA	0.000	−1.618	1.798
	OHIP-14	0.000	1.416	1.215

RT: number of remaining teeth, FTUs: functional tooth units, MP: masticatory performance, HG: handgrip strength, PBA: posterior bite area, ABA: anterior bite area, BF: bite force, MMT: masseter muscle thickness, FIA: food intake ability, TP: tongue pressure.

**Table A2:** Pearson’s correlation coefficient in all subjects.

	Age	RT	FTUs	MP	TP	HG	PBA	ABA	BF	MMT	FIA	OHIP_14
**Age**	1											
**RT**	−0.185 **	1										
**FTUs**	−0.304 ***	0.892 ***	1									
**MP**	0.003	0.079	0.092	1								
**TP**	−0.431 ***	0.132	0.156 *	0.094	1							
**HG**	−0.331 ***	0.157 *	0.155 *	0.099	0.570 ***	1						
**PBA**	−0.279 ***	0.252 ***	0.303 ***	0.306 ***	0.342 ***	0.423 ***	1					
**ABA**	0.039	0.057	0.032	−0.113	−0.041	−0.059	−0.142 *	1				
**BF**	−0.278 ***	0.191 **	0.230 ***	0.287 ***	0.324 ***	0.422 ***	0.870 ***	−0.073	1			
**MMT**	0.035	0.033	0.002	0.193 **	0.314 ***	0.509 ***	0.459 ***	−0.082	0.456 ***	1		
**FIA**	−0.237 ***	0.157 *	0.253 ***	0.170 *	0.260 ***	0.310 ***	0.244 ***	0.025	0.268 ***	0.160 *	1	
**OHIP_14**	0.043	0.009	−0.057	−0.142 *	−0.084	−0.141 *	−0.136 *	0.002	−0.158 *	−0.112	−0.518 ***	1

*p* *** < 0.001, *p* ** < 0.01, *p* * < 0.05. RT: number of remaining teeth, FTUs: functional tooth units, MP: masticatory performance, HG: handgrip strength, PBA: posterior bite area, ABA: anterior bite area, BF: bite force, MMT: masseter muscle thickness, FIA: food intake ability, TP: tongue pressure.
