# Supplementary material for: Assessment of Age-Related Changes on Masticatory Function in a Population with Normal Dentition
Source: Int J Environ Res Public Health. 2021 Jun 27;18(13):6899. doi: 10.3390/ijerph18136899 (PMC8296882; doi:10.3390/ijerph18136899)
Supplement: Supplementary file 1 [file ijerph-18-06899-s001.zip › supplementary files_tables_IJERPH_김선희(최종).pdf]

Table S1. Multiple linear regression analysis of factors associated with masticatory performance

		Univariate					Multivariate				
		B	β	95% CI	*p*	R^2	B	β	95% CI	*p*	R^2
20-45	Age	1.715	0.229	-0.381 - 3.811	0.107	0.052					
	Gender (M)	1		ref							
	Gender (F)	-4.038	-0.037	-35.775 - 27.698	0.799	0.001					
	RT	-6.073	-0.172	-16.034 - 3.889	0.226	0.030					
	FTUs	-6.313	-0.091	-26.163 - 13.537	0.526	0.008					
	TP	-0.958	-0.187	-2.400 - 0.483	0.188	0.035					
	HG	0.575	0.108	-0.944 - 2.094	0.451	0.012					
	PBA	1.297	0.299	0.108 - 2.485	0.033	0.089	1.164	0.268	0.032 - 2.296	0.044*	0.198
	ABA	-0.765	-0.033	-7.496 - 5.967	0.820	0.001					
	BF	0.033	0.261	-0.002 - 0.068	0.064	0.068					
	MTM	3.639	0.174	-2.275 - 9.554	0.222	0.030					
	FIA	2.450	0.128	-2.983 - 7.884	0.369	0.016					
	OHIP-14	-3.144	-0.356	-5.512 - -0.777	0.010	0.127	-2.926	-0.332	-5.229 - -0.623	0.014*	
	Tooth wear	44.296	0.155	-36.497 - 125.089	0.276	0.024					
45-60	Age	0.235	0.014	-4.328 - 4.797	0.918	0.000					
	Gender (M)	1		ref							
	Gender (F)	-26.904	-0.198	-63.879 - 10.071	0.150	0.039					
	RT	19.478	0.301	2.311 - 36.645	0.027	0.091	-1.848	-0.043	-15.495 - 11.800	0.790	0.106
	FTUs	26.444	0.284	1.563 - 51.326	0.038	0.080	1.015	0.018	-16.639 - 18.669	0.910	
	TP	2.303	0.354	0.608 - 3.997	0.009	0.125	-0.170	-0.032	-0.943 - 0.603	0.664	
	HG	1.346	0.171	-0.806 - 3.498	0.215	0.029					
	PBA	2.316	0.412	0.891 - 3.741	0.002	0.170	1.146	0.219	-0.308 - 2.599	0.122	
	ABA	-2.037	-0.176	-5.215 - 1.142	0.204	0.031					
	BF	0.066	0.420	0.026 - 0.106	0.002	0.176	0.012	0.087	-0.026 - 0.051	0.527	
	MTM	8.014	0.311	1.193 - 14.835	0.022	0.097	0.425	0.018	-3.724 - 4.574	0.840	
	FIA	3.212	0.230	-0.572 - 6.995	0.094	0.053					
	OHIP-14	-0.254	-0.030	-2.571 - 2.063	0.827	0.001					
	Tooth wear	23.826	0.125	-28.819 - 76.471	0.368	0.016					
61-70	Age	-1.938	-0.103	-7.712 - 3.836	0.502	0.011					
	Gender (M)	1		ref							
	Gender (F)	-8.696	-0.082	-41.278 - 23.885	0.593	0.007					
	RT	8.009	0.169	-6.326 - 22.344	0.266	0.029					
	FTUs	9.537	0.194	-5.283 - 24.357	0.201	0.038					
	TP	0.261	0.038	-1.845 - 2.368	0.804	0.001					
	HG	0.205	0.036	-1.569 - 1.979	0.817	0.001					
	PBA	1.547	0.301	0.042 - 3.053	0.044	0.091	1.547	0.301	0.042 - 3.053	0.044*	0.091
	ABA	-0.720	-0.233	-1.644 - 0.204	0.124	0.054					
	BF	0.034	0.268	-0.004 - 0.073	0.076	0.072					
	MTM	1.463	0.059	-6.093 - 9.018	0.698	0.004					
	FIA	1.683	0.188	-1.023 - 4.389	0.217	0.035					
	OHIP-14	-0.999	-0.139	-3.185 - 1.187	0.362	0.019					
	Tooth wear	-28.722	-0.265	-60.820 - 3.375	0.078	0.070					
71+	Age	0.385	0.034	-2.523 - 3.294	0.792	0.001					
	Gender (M)	1		ref							
	Gender (F)	-16.427	-0.138	-47.165 - 14.311	0.289	0.019					
	RT	2.717	0.068	-7.721 - 13.156	0.604	0.005					
	FTUs	-0.099	-0.002	-12.260 - 12.061	0.987	0.000					
	TP	0.234	0.039	-1.322 - 1.790	0.764	0.002					

HG	0.613	0.085	-1.270 - 2.495	0.517	0.007
PBA	1.477	0.203	-0.375 - 3.329	0.116	0.041
ABA	1.659	0.106	-2.396 - 5.714	0.416	0.011
BF	0.030	0.161	-0.018 - 0.079	0.217	0.026
MTM	3.097	0.138	-2.677 - 8.872	0.288	0.019
FIA	1.235	0.136	-1.112 - 3.582	0.297	0.018
OHIP-14	-1.113	-0.117	-3.571 - 1.346	0.369	0.014
Tooth wear	13.457	0.103	-20.555 - 47.469	0.432	0.011

B: partial regression coefficient; β: standardized partial regression coefficient; * $p<0.05$
RT: number of remaining teeth, FTUs: functional tooth units, MP: masticatory performance, HG: handgrip strength, PBA: posterior bite area, ABA: anterior bite area, BF: bite force, MMT: masseter muscle thickness

Table S2. Multiple linear regression analysis of factors associated with food intake ability

		Univariate					Multivariate				
		B	β	95% CI	*p*	R^2	B	β	95% CI	*p*	R^2
20-45	Age	0.029	0.073	-0.084 - 0.141	0.609	0.005					
	Gender (M)	1		ref							
	Gender (F)	-1.323	-0.228	-2.943 - 0.297	0.107	0.052					
	RT	-0.178	-0.097	-0.706 - 0.349	0.500	0.009					
	FTUs	-0.395	-0.108	-1.433 - 0.644	0.449	0.012					
	TP	0.018	0.068	-0.058 - 0.095	0.634	0.005					
	HG	0.091	0.327	0.016 - 0.167	0.019	0.107	0.057	0.203	-0.043 - 0.156	0.257	0.329
	PBA	0.044	0.194	-0.020 - 0.108	0.172	0.038					
	ABA	0.188	0.153	-0.161 - 0.536	0.285	0.023					
	BF	0.002	0.229	0.000 - 0.003	0.106	0.053					
	MTM	0.319	0.291	0.017 - 0.620	0.039	0.084	0.198	0.180	-0.194 - 0.589	0.315	
	MP	0.007	0.128	-0.008 - 0.022	0.369	0.016					
	OHIP-14	-0.208	-0.449	-0.326 - -0.089	0.001	0.201	-0.216	-0.468	-0.328 - -0.104	<.001*	
	Tooth wear	-1.796	-0.120	-6.050 - 2.458	0.400	0.014					
45-60	Age	-0.148	-0.126	-0.472 - 0.176	0.365	0.016					
	Gender (M)	1		ref							
	Gender (F)	-0.223	-0.023	-2.922 - 2.477	0.869	0.001					
	RT	1.241	0.268	0.000 - 2.483	0.050	0.072					
	FTUs	2.982	0.447	1.321 - 4.644	0.001	0.200	4.378	0.656	1.758 - 6.997	0.002*	0.447
	TP	0.115	0.248	-0.010 - 0.241	0.071	0.061					
	HG	0.072	0.129	-0.083 - 0.227	0.353	0.017					
	PBA	0.101	0.251	-0.007 - 0.209	0.067	0.063					
	ABA	0.144	0.174	-0.083 - 0.372	0.209	0.030					
	BF	0.004	0.315	0.001 - 0.007	0.020	0.099	0.001	0.107	-0.001 - 0.004	0.356	
	MTM	0.042	0.023	-0.471 - 0.556	0.870	0.001					
	MP	0.016	0.230	-0.003 - 0.036	0.094	0.053					
	OHIP-14	-0.252	-0.422	-0.402 - -0.101	0.001	0.178	-0.262	-0.440	-0.394 - -0.130	<.001*	
	Tooth wear	2.054	0.151	-1.700 - 5.809	0.277	0.023					
61-70	Age	-0.456	-0.216	-1.088 - 0.177	0.154	0.047					
	Gender (M)	1		ref							

	Gender (F)	-2.185	-0.184	-5.771 - 1.402	0.226	0.034					
	RT	1.049	0.199	-0.542 - 2.641	0.191	0.040					
	FTUs	1.247	0.227	-0.395 - 2.889	0.133	0.052					
	TP	0.347	0.454	0.138 - 0.557	0.002	0.206	0.168	0.220	0.002 - 0.335	0.047*	0.691
	HG	0.239	0.370	0.054 - 0.423	0.012	0.137	0.027	0.041	-0.150 - 0.203	0.761	
	PBA	0.080	0.139	-0.095 - 0.254	0.361	0.019					
	ABA	-0.012	-0.036	-0.118 - 0.094	0.816	0.001					
	BF	0.003	0.175	-0.002 - 0.007	0.251	0.031					
	MTM	0.769	0.280	-0.042 - 1.580	0.063	0.078					
	MP	0.021	0.188	-0.013 - 0.055	0.217	0.035					
	OHIP-14	-0.639	-0.797	-0.788 - -0.490	<.001	0.635	-0.573	-0.715	-0.732 - -0.414	<.001*	
	Tooth wear	-0.778	-0.064	-4.486 - 2.931	0.674	0.004					
71+	Age	-0.012	-0.010	-0.332 - 0.308	0.940	0.000					
	Gender (M)	1		ref							
	Gender (F)	-4.209	-0.321	-7.441 - -0.977	0.012	0.103	-3.674	-0.280	-7.627 - 0.279	0.068	0.310
	RT	0.596	0.135	-0.544 - 1.736	0.300	0.018					
	FTUs	0.907	0.177	-0.409 - 2.223	0.173	0.031					
	TP	0.040	0.061	-0.131 - 0.211	0.642	0.004					
	HG	0.219	0.274	0.019 - 0.419	0.032	0.075	-0.020	-0.025	-0.265 - 0.226	0.872	
	PBA	0.182	0.228	-0.021 - 0.384	0.078	0.052					
	ABA	0.218	0.126	-0.227 - 0.663	0.332	0.016					
	BF	0.004	0.208	-0.001 - 0.010	0.108	0.043					
	MTM	0.408	0.166	-0.224 - 1.041	0.201	0.028					
	MP	0.015	0.136	-0.013 - 0.043	0.297	0.018					
	OHIP-14	-0.513	-0.491	-0.751 - -0.276	<.001	0.241	-0.483	-0.462	-0.720 - -0.246	<.001*	
	Tooth wear	-1.260	-0.087	-5.006 - 2.486	0.504	0.008					

B: partial regression coefficient; β: standardized partial regression coefficient; * p<0.05
RT: number of remaining teeth, FTUs: functional tooth units, MP: masticatory performance, HG: handgrip strength, PBA: posterior bite area, ABA: anterior bite area, BF: bite force, MMT: masseter muscle thickness
